# Phosphatic rock weathering and agricultural activities driving the dynamics of potentially toxic elements in surface soil of weathered zone

**DOI:** 10.7717/peerj.21110

**Published:** 2026-04-20

**Authors:** Haiyan Zhang, Changguang Liao, Chao Wang, Xinggang Ye, Bo Du, Ying Yu, Lei Zhang

**Affiliations:** 1 College of Ecology and Environment, Hubei Industrial Polytechnic, Shiyan, China; 2 College of Environment, Hohai University, Nanjing, China; 3 Bioengineering College, Chongqing University, Chongqing, China; 4 China Resources Double-crane Pharmaceutical Co.,Ltd., Beijng, China; 5 Nanjing Institute of Environmental Science, Ministry of Ecology and Environment Peoples Republic of China, Nanjing, China

**Keywords:** Potentially toxic elements, Phosphatic rock, Farmland soil, Weathered profiles, Source analysis

## Abstract

Phosphatic rock weathering and agricultural activities may pollute soils with potentially toxic elements (PTEs) in phosphate-rich regions. However, the behavior of PTEs during rock weathering and their subsequent fate under agricultural influence remain poorly understood. Therefore, the natural weathering profile and farmland soil in a typical phosphatic zone were selected as the subjects for investigating the behavior characteristics of PTE during weathering, as well as PTE bioavailability and sources in surface soil under the influence of agricultural activities. The investigation into natural weathered profiles demonstrated that the weathering of phosphatic rock could generate a significant geological background of cadmium (Cd), lead (Pb) and mercury (Hg) due to their geochemical fractions in minerals. The agricultural activities were associated with elevated levels and increased bioavailability of PTE, particularly lead (Pb), copper (Cu) and zinc (Zn). In farmland topsoil, mean Cd and Pb concentrations reached 0.86 and 151 mg/kg, respectively, with 87% and 70% of samples classified as seriously polluted. The correlation analysis indicated that iron oxide was the predominant controlling factor for the bioavailability of PTE in farmland soil. The results of sources based on Pb isotope and positive matrix factorization (PMF) model suggested that the presence of Cd and Pb in farmland soil was mainly caused by the pesticide use and fertilization, respectively, with PMF attributing approximately 81% and 74% of their totals to these anthropogenic sources. Our study reveals that severe PTE pollution in the phosphatic zone stems from the synergistic effects of natural rock weathering and agricultural activities. This research served as a valuable reference for the prevention and control of PTE in the phosphatic zone.

## Introduction

Phosphatic rock could be associated with large amounts of potentially toxic elements (PTE) during deposition (Gnandi et al., 2006; Mohammad et al., 2014). Faridullah et al. (2017) observed that sedimentary phosphatic rocks displayed an enrichment trend in arsenic (As), cadmium (Cd), chromium (Cr) and lead (Pb). Due to the high solubility and rapid weathering of phosphatic rock, these PTE accumulated in bedrock could enter the supergene environment during soil formation processes, posing a threat to surface ecology (Li et al., 2004). However, the migration and enrichment behavior of PTE during the weathering process of phosphatic rock was poorly understood. Previous studies have predominantly focused on the contamination of PTE resulting from phosphate ore mining and phosphogypsum stacking (Dzagli et al., 2022; Hazou et al., 2021), whereas recent findings suggested that natural bedrock weathering was also a significant contributor to PTE pollution (Qu et al., 2020; Wei et al., 2022). In addition to the alteration in PTE content during weathering, the availability of PTE in soil determined plant uptake and ecological risk, which was strongly influenced by the geochemical fractions of PTE (Yu et al., 2020). The dearth of research on the geochemical fractions of PTE during the weathering process impeded the risk identification of PTE and hindered efforts to prevent and control PTE in phosphoric zone.

It was determined that the farmland soil and crops within the phosphoric zone experienced severe pollution from PTE (Dzagli et al., 2022; Mabrouk, Mabrouk & Mansour, 2020; Tagumira, Biira & Amabayo, 2022). Agricultural activities have been demonstrated to exert a significant impact on the contamination of PTE in surface soil (Chen et al., 2022). However, the specific causes of PTE pollution of farmland soil in phosphoric zone remained unclear and were generally attributed to mining activities, while overlooking the contribution of agricultural activities (Hou et al., 2019; Mohammad et al., 2014). This environmental challenge is not regionally isolated. Parallel studies in global phosphate mining areas, such as the Gafsa-Metlaoui basin in North Africa, have also documented severe PTE contamination (particularly Cd and Pb) and assessed their significant health risks *via* multiple exposure pathways (Khelifi et al., 2021). This underscores the global relevance of PTE pollution in phosphate-rich ecosystems. To effectively manage this widespread issue, a critical prerequisite is to accurately quantify the contributions from different sources within specific regional contexts. Consequently, in order to examine the impact of agricultural activities on PTE of surface soil in phosphatic zone, it is imperative to ascertain the sources of PTE. Meanwhile, comprehending the sources of PTE is crucial in implementing targeted measures to control local PTE pollution. Stable isotope ratios of specific elements (*e.g.*, Pb) are powerful tracers for pollutant source identification, as they often exhibit characteristic signatures (‘fingerprints’) that can distinguish between different anthropogenic and geogenic sources (Bi et al., 2017). Nevertheless, the isotope technology can only trace the origin of a single element, thus necessitating the application of statistical techniques (*e.g.*, positive matrix factorization and principal component analysis-multiple linear regression) (Haghnazar et al., 2022). Positive matrix factorization (PMF) model has been effectively utilized for PTE source apportionment in air, soil, and sediment matrices (Sakizadeh & Zhang, 2021). The proposed method not only enables quantitative analysis of PTE, but also offers the advantages of avoiding source component spectrum measurement, non-negativity constraint on decomposition matrix elements, and utilization of data standard deviations for optimization (Manousakas et al., 2017). Recently, the integration of isotopic fingerprinting (*e.g.*, Pb, zinc (Zn), Cd isotopes) with receptor models like PMF has emerged as a powerful approach to overcome the limitations of single-method tracing, enabling more robust and quantitative discrimination between geogenic and anthropogenic sources (Cao et al., 2025).

Effective management of PTE risks in these regions requires a clear discrimination between geogenic and anthropogenic sources. A critical but underexplored scientific question in such regions is not merely the magnitude of pollution from each source, but their interaction—specifically, how a high natural geogenic background may modulate the environmental fate and risk of anthropogenically introduced PTEs, and *vice versa*. Despite the recognized severity of PTE pollution in phosphate-rich zones (Cheng et al., 2021; Shang et al., 2021), a problem also documented in major mining areas like North Africa (Khelifi et al., 2021), the relative contributions and interactions between the natural weathering flux from phosphate bedrock and anthropogenic inputs from agriculture remain poorly quantified. To address this gap, this study is guided by the following research questions: (1) How do PTEs migrate and enrich during phosphate rock weathering? (2) What are the geochemical fractions and inherent mobility of these PTEs? (3) What factors control the bioavailability of PTEs in farmland soils? (4) What are the quantitative contributions of geogenic *versus* agricultural sources to PTE pollution in topsoil? By integrating investigations of weathering profiles and farmland soils with geochemical, isotopic, and receptor modeling techniques, we seek to answer these questions.

## Materials and Methods

### Study design and prior expectations

This study employed a combined observational and comparative design to investigate PTE dynamics in a phosphate-rich region. Field sampling targeted both natural weathering profiles (to capture geogenic processes) and adjacent farmland topsoil (to assess anthropogenic influence). The primary outcomes were: (i) the mass transfer coefficients and geochemical fractions of PTEs in weathering profiles; (ii) the total concentrations, bioavailability (Mehlich-3 extractable), and pollution indices of PTEs in farmland soils; and (iii) the quantified source contributions to farmland PTEs derived from Positive Matrix Factorization modeling. Our *a priori* expectations, corresponding to the four study objectives, were: (1) that PTEs would exhibit distinct enrichment or depletion patterns (*τ* values) during weathering, dictated by their host minerals; (2) that the geochemical fractions would reveal Cd and Hg to have higher mobile fractions than other PTEs; (3) that soil iron oxide content would be a key negative regulator of PTE bioavailability in farmland; and (4) that Pb isotope ratios and PMF modeling would differentiate between geogenic (bedrock weathering) and anthropogenic (fertilizer/pesticide) sources, with agriculture being the dominant source for surface enrichment of Cd and Pb.

### Study area

The research area is situated in the southern region of Kunming and northern part of Guiyang, which constitutes an important phosphoric zone in southwest China. The study region in southern Kunming is longitude 102.42°∼102.89° East, latitude 24.36°∼24.90° North, belonging to the Kunming-Huaning polyphosphorus basin, which is one of the three major polyphosphorus basins in Yunnan province. The study region in the north of Guiyang is longitude 106.72°∼107.29° East, latitude 26.99°∼27.29° North, which is located in a prominent ore zone abundant in mega-phosphate deposits. The ore-forming characteristics of phosphate ore in the study area are sedimentary type, with the main constituent minerals belonging to the calcium (Ca) and magnesium (Mg) types, which is greatly affected by weathering and leaching.

The study area features a subtropical plateau-type humid monsoon climate, which falls under the agricultural geographical type with plantation as the primary industry. The terrain predominantly comprises of medium to high mountainous hills, characterized by extensive karst topography. The soil composition consists of red, xerothermic soil, lateritic red earth and yellow earth.

### Sample collection and chemical analysis

**Figure 1:**
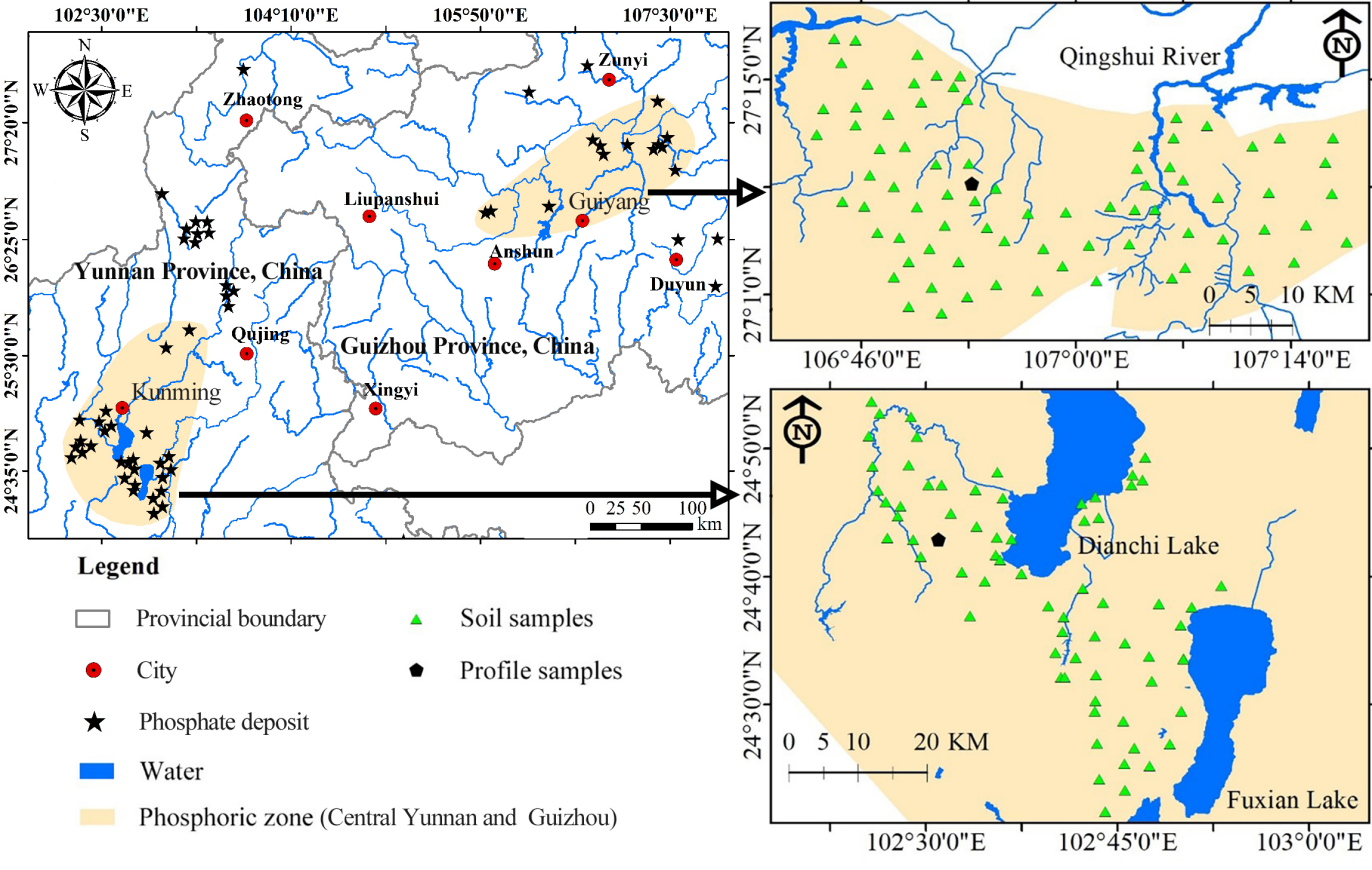
Distribution map of sample points. The location of the study area and the distribution of the sampling locations.

Natural weathered profiles (sampled down to 180 cm depth at 20 cm intervals) and farmland soil samples were collected at 148 sites (two for profiles and 146 for farmland soils) within the survey region. Two representative natural weathering profiles were purposively selected in undisturbed areas, one from each of the two major sub-regions (Kunming and Guiyang). These profiles were not intended for geostatistical representation but to elucidate the *in-situ* geochemical processes and mechanisms of PTE release and transformation during the weathering of the local phosphate bedrock. Their selection was based on clear exposure, minimal anthropogenic disturbance, and representation of the dominant bedrock type in each area. Farmland soil samples (0–20 cm depth, the plow layer most affected by agricultural activities) were collected using a stratified random strategy. The study area was first stratified based on land-use type (paddy field, dryland) and proximity to the phosphate bedrock outcrops. Within each stratum, sample locations were randomly distributed to ensure coverage of the spatial variability of PTE concentrations. This design aimed to capture the regional-scale contamination status and its association with agricultural land use, rather than point-source pollution. A differential global positioning system (GPS) was used to record the locations of sampling (Fig. 1).

**Figure 2:**
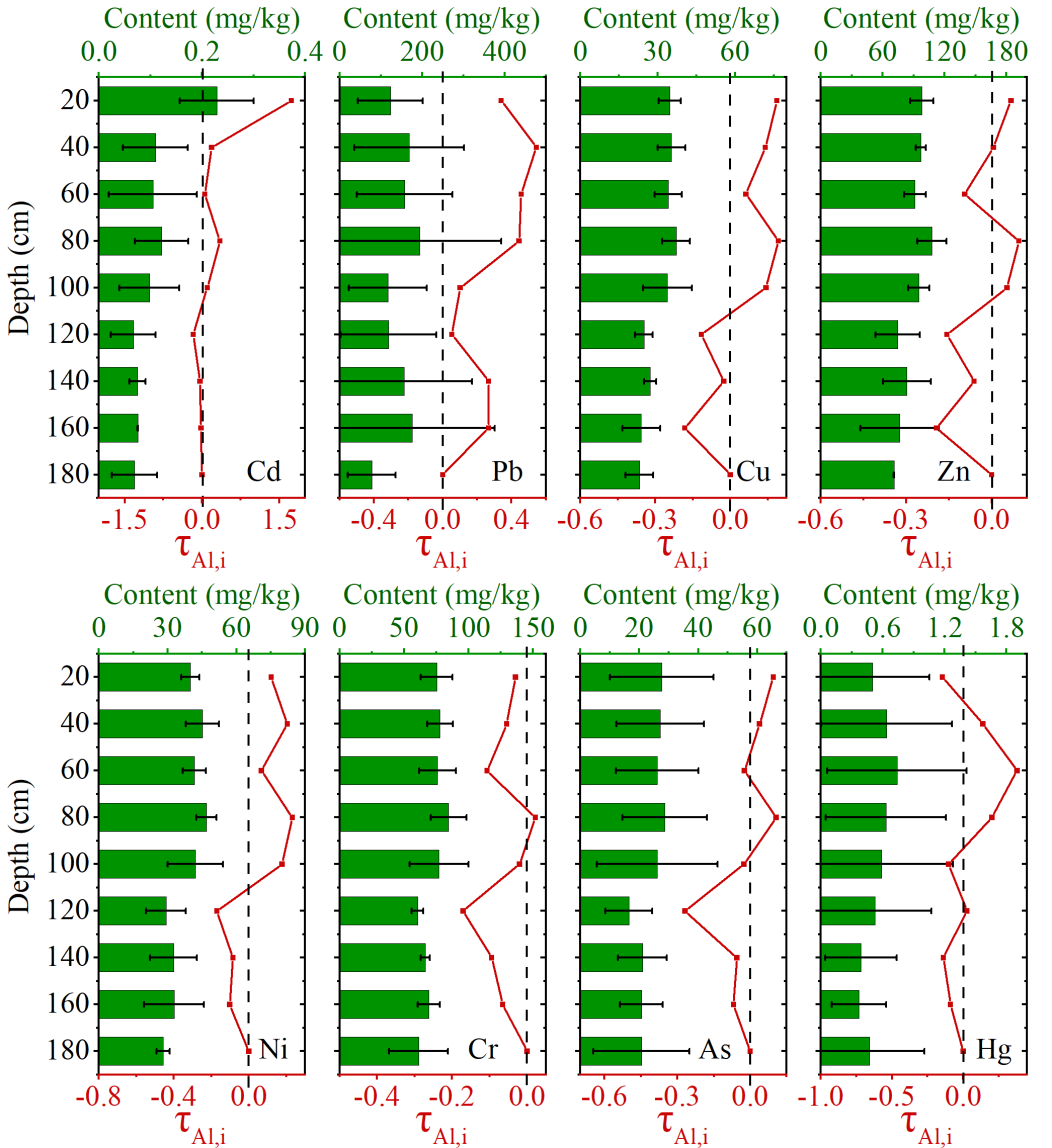
PTE concentration distribution and mass balance coefficient “*τ* ” in weathered profiles (combined data from two sites). The concentration distribution, while the line graph depicts the enrichment or depletion (*τ* > 0 or *τ* < 0) of each element relative to the parent bedrock (*n* = 9 for each profile layer). Error bars represent ±1 standard deviation (SD).

**Figure 3:**
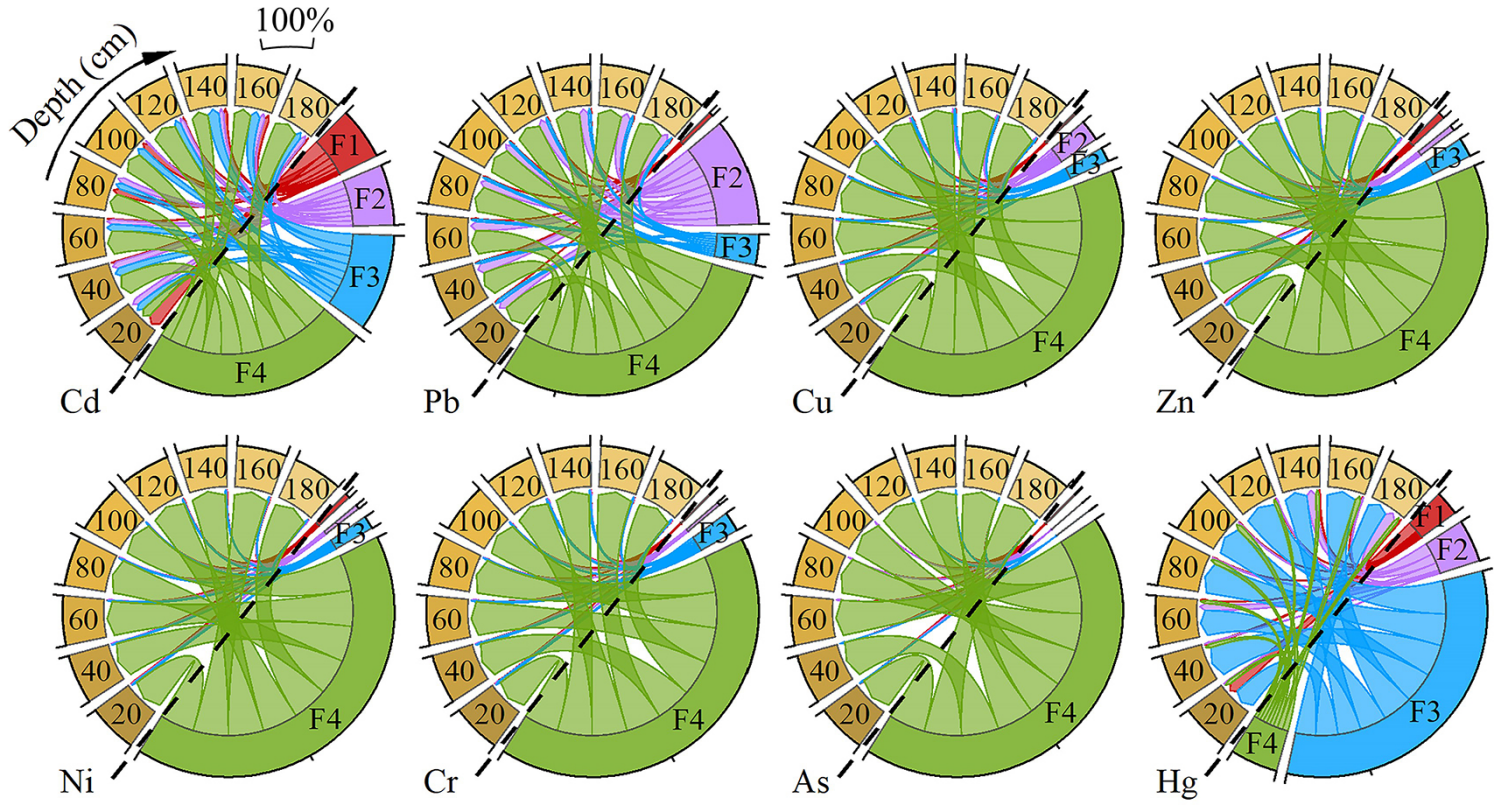
Geochemical fractions of PTE in the weathered profiles (combined data from two sites). The proportion of potentially toxic elements at different depths of the weathering profile. F1 represents the weak acid soluble fraction; F2 represents the reducible fraction; F3 represents the oxidizable fraction; F4 represents the residual fraction (*n* = 9 for each profile layer).

All soil samples (profiles and farmland) were air-dried at ambient temperature and then ground. The <2 mm fraction, obtained by sieving through a nylon sieve, was used for the analysis of general soil physicochemical properties and major elements. Some of the soil samples were ground to pass through 100 meshes and stored in closed polyethylene bags for PTE content analysis. Given that the primary objective was to investigate the general behavior (enrichment/depletion patterns and fractionation) of PTEs during phosphate rock weathering, and because preliminary analysis showed similar geochemical trends between the two profiles, the data from both profiles were combined for a synthesized presentation in Figs. 2 and 3 to illustrate the overarching weathering dynamics.

The soil pH was determined by an acidity meter (pH-3C, China) with a soil/water ratio of 1:2.5 (*W/V*) (Dai et al., 2017). The cation exchange capacity (CEC) was determined with the sodium acetate-flame photometric method, as described in Text S1. Soil organic matter (SOM) was determined with the potassium dichromate-concentrated method with sulfuric acid external heating. The determination of major elements was performed by X-ray fluorescence spectroscopy (SUPER XRF 2400, China).

PTE (Cd, Pb, Cu, Zn, nickel (Ni), Cr, arsenic (As) and mercury (Hg)) in soil samples were measured as follows. Soil samples were digested with a mixture of HF, HNO_3_ and HClO_4_ (4:5:2, *v*/*v*/*v*) for the determination of total PTE contents (Liu et al., 2017). The treatment of PTE geochemical fractions (weak acid soluble fraction, reducible fraction, oxidizable fraction and residue fraction) in soil was described in Text S2. The bioavailable state of PTE in soil was extracted by Mehlich3 (M3) solution, which was a mixture of 0.2 M CH_3_COOH, 0.25 M NH_4_NO_3_, 0.015 M NH_4_F, 0.013 M HNO_3_, and 0.001 M EDTA (Mehlich, 1984). The determination of Cd, Pb, Cu, Zn, Ni and Cr was performed by inductively coupled plasma coupled plasma-mass spectrometry (IRIS Advantage, Thermo Jarrell Ash Co., USA), and the concentrations of Hg and As were measured by atomic fluorescence spectrometry (AFS-2100, China) following hydride generation. For these elements, soil samples were digested with aqua regia (HCl: HNO_3_ = 3:1, *v/v*) prior to analysis, in accordance with Chinese national standard method HJ 680-2013.

The two representative weathering profiles were selected in undisturbed areas to reflect the natural weathering sequence of the local phosphate bedrock. The 146 farmland sites were distributed using a stratified random sampling strategy across the main agricultural land-use types (*e.g.*, paddy fields, dry land) within the phosphate zone, avoiding obvious point sources of pollution (*e.g.*, villages, roads, industrial sites). No samples were excluded during field collection. All collected samples (*n* = 164 for profiles and farmland topsoil combined) passed laboratory quality control procedures and were included in the subsequent analyses. After quality control, the final sample numbers used in the different analyses were as follows: (i) soil physicochemical properties and total PTE concentrations: *n* = 164 (146 topsoil + 18 profile samples); (ii) geochemical fractionation: *n* = 18 (profile samples only); (iii) Mehlich-3 extractable (bioavailable) PTE concentrations: *n* = 146 (topsoil samples only); (iv) Pb isotopic analysis: *n* = 9 (3 bedrock + 6 topsoil samples); (v) Positive Matrix Factorization (PMF) source apportionment: based on the PTE concentration matrix of the *n* = 146 topsoil samples. The sample size for the farmland topsoil (*n* = 146) was determined to achieve a representative characterization of PTE contamination at the regional scale. This sampling density is consistent with, or exceeds, that employed in similar geochemical baseline studies in karst regions (Hu et al., 2024). It provides sufficient statistical power to identify major spatial trends and source contributions using receptor modeling (*e.g.*, PMF). Detailed procedures for sequential extraction, Mehlich-3 extraction, and comprehensive quality assurance/quality control (QA/QC) measures are provided in Texts S2, S3, and S4, respectively.

### Determination of Pb isotopes

Fresh, unweathered phosphatic bedrock samples (*n* = 3) were collected from the bottom of the weathering profiles and/or from nearby outcrops within the study area to represent the parent material. Phosphatic bedrock and farmland soil powder samples were then decomposed by high-pressure PTFE bombs for Pb isotope analysis. Phosphatic bedrock and farmland soil powder samples were decomposed by high-pressure PTFE bombs. The first exchange column combined with BioRad AG50W × 8 and Sr Spec resin was used to separate Pb from sample matrix following established chromatographic procedures (*e.g.*, Weis et al., 2006). The Pb-bearing elution were dried down and re-dissolved in 1.0 ml 2wt% HNO_3_. Small aliquots of each were analyzed using Agilent Technologies *7700x* quadrupole ICP-MS (Hachioji, Tokyo, Japan) to determine the exact contents of Pb available. Diluted solution (40ppb Pb doping with 10 ppb Tl) were introduced into Nu Instruments *Nu Plasma II* MC-ICP-MS (Wrexham, Wales, UK) by Teledyne Cetac Technologies *Aridus II* desolvating nebulizer system (Omaha, Nebraska, USA). Raw data of isotopic ratios were corrected for mass fractionation by normalizing to ^205^Tl/^203^Tl = 2.3885 for Pb with exponential law. Geochemical reference materials of USGS BCR-2, BHVO-2, AVG-2, RGM-2 were treated as quality control. The measured Pb isotope ratio was within the error range reported by Weis et al. (2006).

### Mass transfer coefficient

In order to explore the migration behavior of PTE during phosphatic rock weathering, mass migration coefficient (MTC) of elements from bedrock to soil was used to evaluate the enrichment trend of various PTE in weathered profiles. MTC is a deterministic geochemical index calculated from elemental concentration ratios. While it does not have an ‘error bar’ in the statistical sense, its value reflects the net enrichment or depletion resulting from weathering processes, and its trend across the profile is the primary interpretative focus. MTC is capable of effectively quantifying the profit and loss of PTE in relation to bedrock, which is represented by “*τ*”, the calculation formula is as follows (Sun et al., 2022).

(1) \begin{eqnarray*}{\tau }_{i,j}= \frac{{C}_{i,s}{C}_{j,b}}{{C}_{j,s}{C}_{i,b}} -1\end{eqnarray*}

where C is the concentration of PTE, and the subscripts s and b represent weathered samples and bedrock, respectively. i and j represent the PTE and reference element studied respectively. Al element, due to its relative stability during weathering, can serve as a reference element for normalizing the concentration of PTE (Luo et al., 2015). When the “*τ*” value is less than 0, it reflects the loss of PTE relative to the bedrock in the weathering process; when the “*τ*” value is more than 0, it reflects the enrichment of PTE relative to the bedrock in the weathering process.

### Assessment of PTE pollution in farmland soils

In order to assess the PTE pollution of farmland soil in phosphoric zone, the single pollution index (*P*_*i*_) was adopted. *P*_*i*_ has been commonly used to indicate pollution level of PTE in ecological risk assessment, the calculation is as follows (Zhang et al., 2018).

(2) \begin{eqnarray*}{P}_{i}={C}_{i}/{B}_{i}\end{eqnarray*}

where C is the concentration of PTE in farmland soil on phosphoric zone; B is the background value of PTE in farmland surface soil in China; i is the potentially toxic element studied. The classification is shown in Table S1.

### Positive matrix factorization model

In this study, the positive matrix factorization (PMF) model was used to quantify the relative contributions of different sources to PTE pollution in farmland soil within the phosphatic zone. The core of the algorithms for PMF model is to minimize the objective function Q, which is calculated as follows (Guan et al., 2018).

(3) \begin{eqnarray*}{X}_{ij}& =\sum _{k=1}^{p}{g}_{ik}{f}_{kj}+{e}_{ij}\end{eqnarray*}

(4) \begin{eqnarray*}\mathrm{Q}& =\sum _{i=1}^{n}\sum _{j=1}^{m}{ \left( \frac{{x}_{ij}-\sum _{k=1}^{p}{g}_{ik}{f}_{kj}}{{u}_{ij}} \right) }^{2}\end{eqnarray*}

(5) \begin{eqnarray*}\mathrm{c}\leq \mathrm{MDL}~(\text{Method Detection Limit}),{u}_{ij}= \frac{5}{6} ~\mathrm{MDL}\end{eqnarray*}

(6) \begin{eqnarray*}\mathrm{c}> \mathrm{MDL},{u}_{ij}=\sqrt{{ \left( \text{Error fraction}\times \mathrm{c} \right) }^{2}+{ \left( \frac{\mathrm{MDL}}{2} \right) }^{2}}\end{eqnarray*}

where *i*, *j*, *k* are the individual sample, element, source respectively, and *n*, *m*, *p* are total number of samples, elements and sources respectively. *X*_*ij*_ is the concentration of element *j* in sample *i* (mg kg^−1^); *g*_*ik*_ is the contribution amount of source *k* to sample *i* (mg kg^−1^); *f*_*kj*_ is the contribution amount of source *k* to element *j*; c is the concentration of the element in sample; *e*_*ij*_ is the error value of the measured concentration and the concentration predicted by the principal component;*u*_*ij*_ is the uncertainty of element *j* in sample *i*; MDL is the element-specific method detection limit; *Error fraction* is relative standard deviation.

### Statistical analysis

General statistical works were performed using Office 2021 and Origin 2022. ArcGIS10.7 was used to draw the sampling map. Correlation network diagram was obtained by Gephi 0.9.3. PMF analysis results were obtained by EPA PMF V5.0 (USEPA, USA). The results of significance of differences were based on Kruskal–Wallis test (*P* < 0.05). Prior to parametric correlation analysis, the normality of the data distribution for key variables was assessed using the Shapiro–Wilk test and Q-Q plots. Where data deviated significantly from normality, non-parametric Spearman correlation analysis was employed, as reported. Potential outliers were identified using boxplots and Cook’s distance but were retained in the analysis as they represented valid field observations.

## Results and Discussion

### Migration and transformation of PTE during the weathering process of phosphatic rock

#### PTE concentrations in the profiles

Figure 2 presents the combined concentration distributions and mass transfer coefficients (*τ*) of PTEs from both weathering profiles. In weathered profiles, the mean distribution of Cd was in the range of 0.07−0.23 mg/kg, and the mass balance coefficient “*τ*” based on Al as a reference element showed the value of *τ*_Cd_ ranged from −0.17 to 1.7. The content of Cd in the bedrock was not high (Table S2), but significant enrichment was recorded during the weathering process, indicating that Cd accumulated in the surface soil mainly through secondary enrichment. According to relevant study, the mechanism of Cd enrichment in phosphatic bedrock was mainly through cation diffusion (Ibhi, 2014; Zou et al., 2023). Crystal structure of apatite was conducive to the entry of Cd^2+^ (Gnandi et al., 2006). Meanwhile, due to the isomorphism of Cd^2+^ ions and Ca^2+^, Cd^2+^ entering mineral crystals could exchange Ca^2+^, thus remaining (Mohammad et al., 2014). The phosphatic rock in this study mainly consisted of Ca and Mg type phosphatic rock. Consequently, the Cd retained in the initial stage of the rock was unstable. With the enhancement of weathering, a large amount of Ca ions was lost (Fig. S1), which may have provided more binding sites for Cd and contributed to its significant enrichment in topsoil. The Pb ranged from 78.1–194 mg/kg in the profiles, and the value of *τ*_Pb_ was in the range of 0 to 0.54. In the entire weathering profile, there was no loss of Pb element relative to the bedrock. The enrichment of Pb in phosphatic bedrock was dominated by the dissolution/precipitation mechanism, which could form a new mineral phase with a stable hexagonal structure (Ibhi, 2014). Therefore, the overall accumulation trend of Pb was also obvious during weathering. In addition to isomorphic replacement and stable mineral phase formation, surface adsorption of iron and manganese oxides and clay minerals also played an important role in the enrichment of PTE in phosphatic rock (Gnandi et al., 2006). The values of *τ*_Cu_, *τ*_Zn_, *τ*_Ni_, *τ*_Cr_ and *τ*_As_ were in the range of −0.18–0.19, −0.19–0.09, −0.17–0.23, −0.17–0.02 and −0.27–0.11, respectively. The relatively consistent behavior of Cu, Zn, Ni, Cr and As was found in weathered profiles (Fig. 2), which showed behavior consistent with elements known to be adsorbed by iron oxides and clay minerals, as supported by the significant correlations observed in farmland soils (Table S3) and extensive literature (Shi et al., 2025). The value of *τ*_Hg_ ranged from −0.15 to 0.38. The performance of Hg in the profiles was obviously different from other PTE (Fig. 2), indicating that the enrichment method of Hg in phosphatic rock was unique. It is necessary to further explore the geochemical fractions of PTE in the profiles.

It was determined that the surface soil exhibited significant enrichment of Cd and Pb in comparison to the bedrock, while Cu, Zn, Ni and As were only slightly enriched. Conversely, Cr and Hg were found to be deficient. The loss of active calcium and the correlation with iron and aluminum oxides (indicated by the strong negative correlation between TFe_2_O_3_/Al_2_O_3_ and PTE bioavailability in ‘Bioavailability of PTE in farmland soil’) suggests that Fe/Al (hydr)oxides likely play a key role in the retention and enrichment of Cd and Pb during weathering during phosphatic rock weathering, which differed from the Cr accumulation regulated by Fe-(hydro)oxides during basalt weathering and the Cu enrichment induced by carboxyl adsorption during black shale weathering (Derkowski & Marynowski, 2018; Sun et al., 2022).

#### Geochemical fractions of PTE in the profiles

The weathering characteristics of PTE were closely related to their own activation and geochemical fractions (Zhou et al., 2025). The vertical distribution of geochemical fractions as a percentage from total concentrations was illustrated in the Fig. 3. In the process of weathering, the F1 and F2 fractions of Cd tended to accumulate towards the surface, and the F3 fraction was relatively stable, while the F4 fraction showed a deficit. According to the results of PTE fractions proportion, Cd had more likely potential mobility with the highest proportion of F1 than the other seven elements in the weathered profiles, especially in the topsoil, the proportion of F1 accounted for 37%. This result indicates that the natural weathering of phosphatic bedrock releases Cd into the soil environment in a highly mobile form (F1 fraction), thereby creating a significant potential source and elevated geochemical background for Cd contamination in surface soils. The subsequent high bioavailability of Cd measured in the farmland soils (‘Bioavailability of PTE in farmland soil’) confirms that a readily plant-available Cd pool is present in the region, which is consistent with the high mobility observed in the weathering profiles.

The variation of the four fractions of Pb throughout the entire profile was not obvious, which meant that the occurrence form of Pb was relatively stable in the weathering process of phosphatic rock. What’s more, the F2 fraction of Pb was prominent in the profiles, with the reducible fraction (F2) being prominent, a phase often associated with metals bound to Fe-Mn (hydr)oxides, suggesting that such phases likely contribute to Pb retention in these profiles (Yu et al., 2025).

The geochemical fractions of Cu, Zn, Ni, Cr and As in the profiles were pretty stable, mainly existing as F4 fraction (>90%), which signified that these PTE from natural weathering of bedrock posed a low threat to the epigenetic environment.

The geochemical fractions of Hg in the profiles were different from other PTE, with F3 as the main fraction. During weathering process, F1 fraction had an obvious enrichment trend in the topsoil (accounted for 29%), while F2 fraction showed a loss. F3 fraction was significantly enriched in the middle section of the profiles, and F4 fraction remained stable. This result indicated that Hg occurred mainly in the oxidizable fraction in phosphatic bedrock, while other PTE were mainly composed of F4 fraction, which could be used to explain why the behavior of Hg in the profiles was different from other PTE (Fig. 2). The observed depletion of the oxidizable fraction (F3) of Hg in the topsoil, coupled with its enrichment in the middle section (Fig. 3), suggests that under surface oxidizing conditions, this fraction may become mobilized, potentially contributing to the downward migration or loss of Hg from the surface layer.

### Effects of agricultural activities on PTE in surface soil in the phosphatic zone

#### Soil properties and PTE concentrations in farmland soil

Human activities significantly alter PTE concentrations in surface soils. In order to explore the effect of anthropogenic agricultural activities on PTE of surface soil in the phosphoric zone, the soil properties and PTE concentrations in farmland soil were analyzed in Table 1. The mean value of pH was 6.85, showing weak acidity. The SOM and CEC were 2.84% and 20 cmol/kg on average, respectively. The P_2_O_5_ content was 1.67% (0.1%–19.9%), with a high degree of spatial variation (*CV* = 197%). The heterogeneous distribution of P_2_O_5_ likely results from varied fertilization practices and inherent differences in bedrock phosphorus content. The mean contents of total iron oxide (expressed as TFe_2_O_3_), Al_2_O_3_ and SiO_2_ were 5.47%, 19.9% and 53.4% respectively, among which iron and aluminum oxides represented obvious enrichment in farmland soil.

**Table 1:** Statistics of physicochemical properties and PTE contents in the farmland soils (mg/kg dry wt. *n* = 146).

Soil characteristics/PTE	Minimum value	Maximum value	Mean value	Standard deviation	*CV* (%)	Background value^a^,^b^	*P*_*i*_				
							Mean value	Clean	Slightly polluted	Moderately polluted	Seriously polluted
pH	4.44	8.73	6.85	0.93	14	6.7	/	/	/	/	/
SOM (%)	0.42	22.4	2.84	1.97	70	3.1	/	/	/	/	/
CEC (cmol/kg)	7.11	41.0	20.0	8.15	41	/	/	/	/	/	/
P_2_O_5_ (%)	0.10	19.9	1.67	3.29	197	0.18	/	/	/	/	/
Al_2_O_3_ (%)	1.11	68.1	19.9	9.29	47	12.5	/	/	/	/	/
TFe_2_O_3_ (%)	0.20	10.0	5.47	2.23	41	4.2	/	/	/	/	/
SiO_2_ (%)	24.6	105	53.4	15.0	28	/	/	/	/	/	/
Cd	0.02	2.78	0.86	0.61	71	0.097	8.90	1%	4%	8%	**87%**
Pb	1.67	395	151	93.0	61	26	5.82	3%	15%	12%	**70%**
Cu	3.19	127	26.1	19.5	75	22.6	1.16	**58%**	31%	7%	4%
Zn	38.4	523	106	65.4	61	74.2	1.43	34%	**51%**	11%	4%
Ni	0.34	212	56.2	33.1	59	26.9	2.09	10%	**49%**	23%	18%
Cr	15.4	535	137	76.9	56	61	2.25	11%	**36%**	33%	20%
As	4.53	102	21.0	17.9	85	11.2	1.87	32%	**45%**	3%	20%
Hg	0	1.99	0.15	0.29	193	0.065	2.34	**37%**	27%	24%	12%

**Notes.**

*CV*, Coefficient of variation.

a Zhao et al. (2018).

b CNEMC (China National Environmental Monitoring Centre) (1990).

The mean value of pH was 6.85, showing weak acidity. The SOM and CEC were 2.84% and 20 cmol/kg on average, respectively. The P_2_O_5_ content was 1.67% (0.1%–19.9%), with a high degree of spatial variation (CV = 197%). The mean contents of TFe_2_O_3_, Al_2_O_3_ and SiO_2_ were 5.47%, 19.9% and 53.4% respectively, among which iron and aluminum oxides represented obvious enrichment in farmland soil. The bold values indicate the pollution level in which the majority of samples fall for each potentially toxic element (*i.e.,* the mode of the *P*_*i*_ distribution).

The concentrations of Cd and Pb in soil were 0.86 mg/kg (0.02–2.78 mg/kg) and 151 mg/kg (1.67–395 mg/kg) respectively, with 87% (for Cd) and 70% (for Pb) of the soil samples were at seriously polluted levels, showing a high risk. This result indicates that agricultural activities are a major contributor to PTE pollution in the topsoil of the phosphatic zone. However, the specific causes of Cd and Pb pollution in the research area need to be further explored. The average contents of Zn, Ni, Cr, and As were 106 mg/kg, 56.2 mg/kg, 137 mg/kg, and 21 mg/kg, respectively, which were mainly at slightly polluted levels. The majority of soil samples contained Cu and Hg at a clean level, but the *CV* of Hg reached 193%, meaning that there was a high Hg distribution in certain areas. The pollution assessment above relied solely on total PTE concentrations, overlooking bioavailability. This approach may underestimate the ecological risk posed by certain PTEs.

#### Bioavailability of PTE in farmland soil

**Figure 4:**
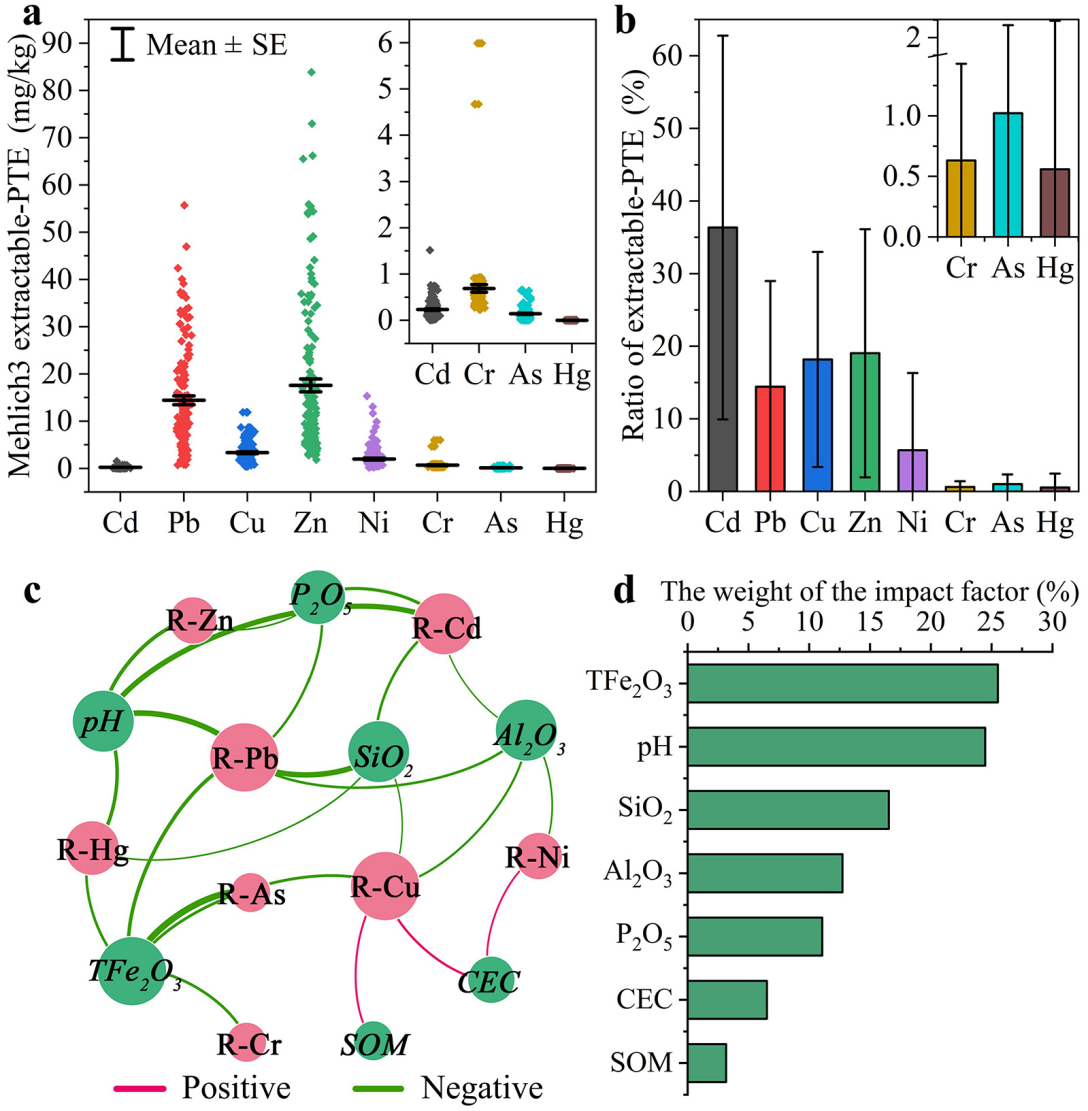
Analysis of bioavailability and impact factors of PTE in farmland soil. (A) Mehlich3 extractable content of PTE; (B) Ratio of Mehlich3 extractable content to total content of PTE; (C) Correlation between soil properties and ratio of Mehlich3 extractable-PTE; (D) The weight of the impact factors (soil properties) (*n* = 146). Error bars represent ±1 standard deviation (SD).

The bioavailability of PTE could better reflect their potential harm to crops. The Mehlich3 (M3) extraction state of PTE provided effective information on the biological availability of PTE (Mehlich, 1984). Figures 4A, 4B showed the M3 extractable content of PTE and corresponding ratio to total content in farmland soil. The PTE of concern were Cd, Pb, Cu, and Zn, with an average ratio of 36.4%, 14.4%, 18.2%, and 19% to total concentrations respectively. These results indicate that agricultural practices lead to higher concentrations of bioavailable PTEs, particularly Cu and Zn, in the soil. Studies have shown that the rhizosphere environment was conducive to enhancing the bioactivity of essential nutrients Cu and Zn for crops, which was inseparable from the specific exudates of crop roots (Jia et al., 2022; Mench & Martin, 1991). Furthermore, the bioavailability of PTE could be significantly affected by soil properties (*e.g.*, pH, soil organic matter and cation exchange capacity, *etc*.) (Houben, Evrard & Sonnet, 2013; Imoto & Yasutaka, 2020). Thus, the correlation between soil properties and M3 extraction rate of PTE were analyzed to explore the effect of soil properties on the bioavailability of PTE in the farmland soil, as shown in Fig. 4C and Table S4. The results showed that soil properties were correlated with the bioavailability of PTE, with TFe_2_O_3_ showing the strongest (negative) association, followed by pH > SiO_2_ > Al_2_O_3_ > P_2_O_5_ >  CEC > SOM (Fig. 4D). The predominance of iron oxide (TFe_2_O_3_) as the key controlling factor for PTE bioavailability is robustly supported by the multivariate correlation network analysis (Fig. 4D), where it exhibited the strongest (negative) association with the extractable fractions of multiple PTEs. This finding is further reinforced by the significant pairwise correlations presented in Table S3. The specific correlations for key influencing factors were discussed below.

##### TFe_2_O_3_.

There was an extremely significant negative correlation between TFe_2_O_3_ and the M3 extraction rates of Pb, Cu, Cr, As and Hg (*P* < 0.01). Iron (hydr)oxides are typically the main adsorbents for PTEs in soil, capable of adsorbing and immobilizing various PTEs through incorporation into or surface complexation with phases such as goethite, ferrihydrite, or other iron (oxyhydr)oxides (Proust et al., 2011; Yan et al., 2020). In addition, iron could serve as a strong reducing agent for certain heavy metal ions such as Cr^6+^, making them solidify in soil with a more stable form of Cr^3+^ (Liu et al., 2022).

##### pH.

H^+^ could activate PTE and reduce the adsorption of clay minerals and silicate minerals on heavy metal ions (Loganathan et al., 2012). Therefore, pH showed an extremely significant negative correlation with M3 extraction rates of Cd, Pb, Zn, and Hg (*P* < 0.01) in farmland soil.

##### SiO_2_.

Silicon could be co-precipitated with PTE by forming complexes and silica-hemicellulose matrix, leading to the decline of the activated PTE in soil (Zhao et al., 2022a; Zhao et al., 2022b). In this study, silicon mainly acted on Cd and Pb, showing an extremely significant negative correlation with M3 extraction rates of them (*P* < 0.01).

Probing the main factors affecting the bioavailability of PTE in farmland soil on the phosphatic zone was beneficial to provide treatment strategies for PTE pollution in the region. Given the exploratory nature of the correlation analysis aimed at identifying potential controlling factors for bioavailability, and to avoid overly conservative Type II errors, we reported uncorrected *p*-values while interpreting results with caution, focusing on patterns with *P* <  0.01 as more robust.

### Sources of PTE in farmland soil

#### Pb isotopes

**Figure 5:**
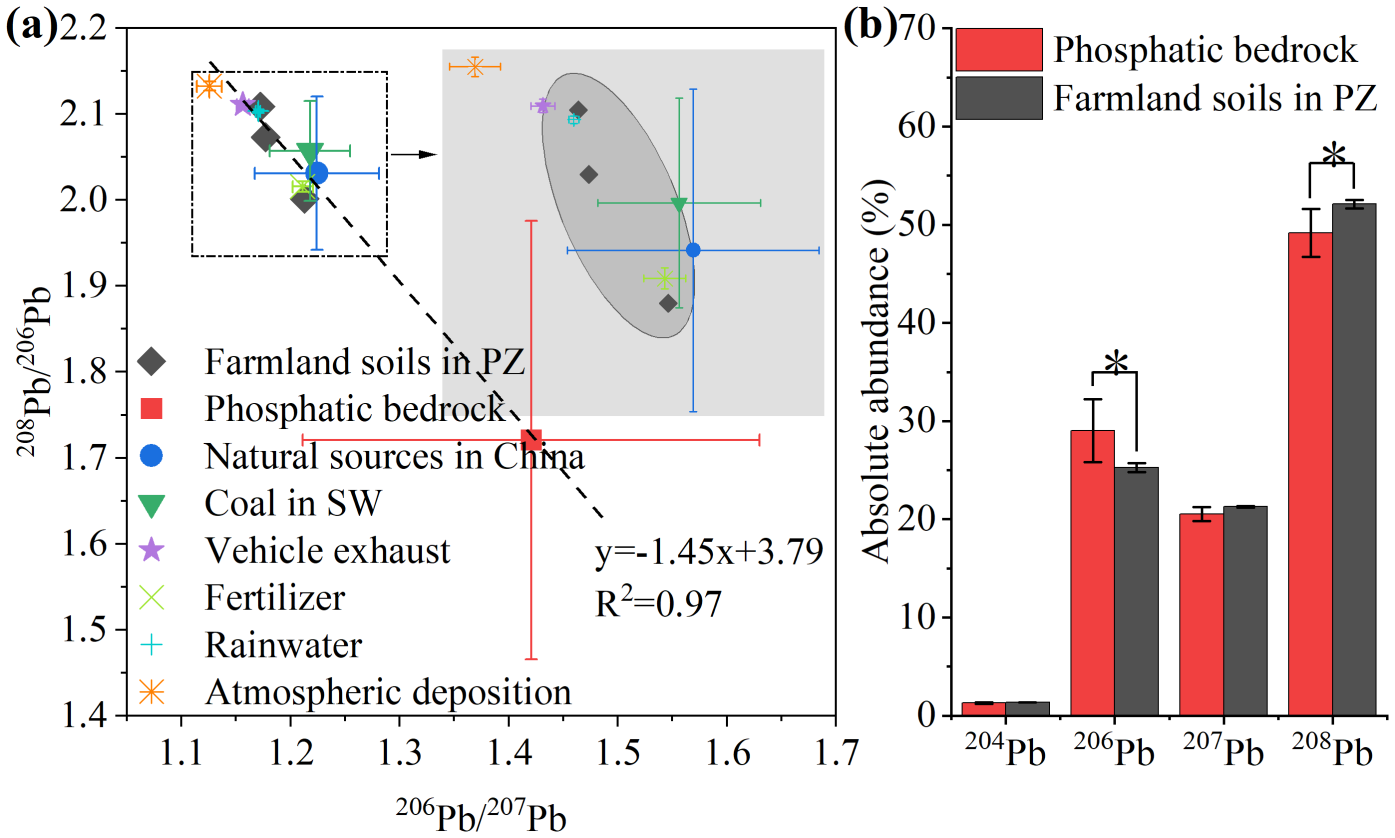
Pb isotope compositions of farmland soils on phosphatic zone (PZ) and end member. The asterisk “*” indicates significant differences of Pb isotope compositions between phosphatic bedrock and farmland soil in PZ (*P* < 0.05) (*n* = 9). Error bars representing the 2*σ* external reproducibility. Reference data for potential end-members (natural sources, coal, fertilizer, *etc*.) are from Bi et al. (2017), Zhao et al. (2015), and Liu et al. (2019).

In order to explore the characteristics of Pb in farmland soil on phosphatic zone, Pb isotopes in phosphatic bedrock samples and farmland soil samples were analyzed, and Pb isotope compositions of end member that might have an impact on farmland soil were collected, as shown in the Fig. 5 and Table S5. The ^206^Pb/^207^Pb ratios of phosphatic bedrock ranged from 1.253 to 1.716, and the ^208^Pb/^206^Pb ratios ranged from 1.362 to 1.928. The Pb isotope ratios of phosphatic bedrock varied greatly among the different samples at the study sites. This result indicated that the enrichment of Pb varied in different phosphatic rocks. Faridullah et al. (2017) also found significant differences in the accumulation of PTE in phosphatic rock. The ^206^Pb/^207^Pb and the ^208^Pb/^206^Pb ratios in farmland soil were the range of 1.172–1.213 and 2.002–2.109, respectively. The Pb isotopic composition of farmland soil was significantly different from that of phosphatic bedrock, but similar to that of natural sources in China, coal in SW and fertilizer (Fig. 5A). The result indicated that the similarity of Pb isotopic compositions in farmland soils to those reported for fertilizers, probably phosphate fertilizers, strongly indicates that agricultural fertilization is a major anthropogenic input pathway for Pb. This is consistent with the known occurrence of Pb as a trace impurity in phosphate rocks, which is subsequently transferred to phosphate fertilizers during manufacturing. Studies of Chinese phosphate rocks and fertilizers have documented that they can carry a distinct, often more radiogenic, Pb isotopic signature compared to other anthropogenic sources (Wang et al., 2023). The shift in Pb isotope ratios observed in our topsoil towards the fertilizer end-member field supports this interpretation. In addition, the light isotope ^206^Pb was obviously enriched in bedrock but lost in farmland soil, while the result of heavy isotope ^208^Pb was the opposite (Fig. 5B), further indicating the effect of anthropogenic activities on PTE in the surface soil of the phosphatic zone (Kong et al., 2018).

#### Source apportionment based on PMF model

**Figure 6:**
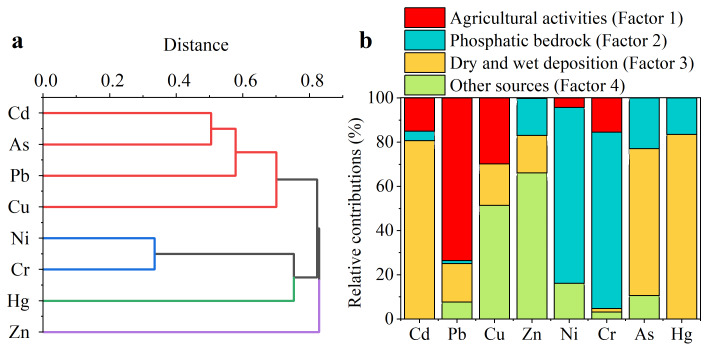
Source apportionment of potentially toxic elements in farmland soil. (A) Hierarchical dendrogram for PTE (each distance reflects the degree of correlation between PTE). (B) Relative contributions of different sources to the PTE in farmland soil on PZ (*n* = 146).

The results of Pb isotope indicated that fertilization had a great contribution to the Pb input of the topsoil in the phosphatic zone. In order to better investigate the sources of PTE of farmland soil in study area, cluster analysis (CA) of PTE in soil samples was conducted first (Fig. 6A, the Pearson correlation coefficients were shown in Fig. S1). PTE with strong correlations were more likely to have the same sources. The result of CA indicated the sources of Cd and As, Ni and Cr in farmland soil were similar. There was a clear distance difference between the cluster 2 (Ni and Cr) and cluster 3 (Hg), suggesting that the PTE in farmland soil had more than two sources.

The relative contributions of potential sources of PTE to the farmland soil were explored further by performing PMF (Q_robust_/Q_exp_ = 0.85). Combined with the results of CA and Pb isotopes above, four factors were set up in the PMF analysis. The base bootstrap runs results including uncertainty was displayed in Table S6. As a whole, contribution of the base runs to the most elements were constrained in the interquartile range (25th–75th percentile) obtained through bootstrap indicating the robustness of base run results. In summary, the results of PMF model were robust and reliable.

The integration of Pb isotope data with PMF results provides a coherent source narrative: the Pb isotopic signature of the topsoil closely aligns with the fertilizer end-member (Fig. 5), which independently validates the interpretation of PMF Factor 1 as a phosphate fertilizer source. Conversely, the distinct isotopic composition of local bedrock supports the attribution of PMF Factor 2 to geogenic weathering. This congruence between the isotopic tracer and the receptor model significantly strengthens the overall source attribution. As shown in Fig. 6B, the characteristic pollutant of Factor 1 was Pb, accounting for 73.62%. According to the Pb isotope composition of farmland soil above, the Pb in the study area was primarily imported from fertilizer (Fig. S2). Javied et al. (2023) also found that phosphorus fertilizers had an obvious effect on Pb accumulation in farmland soil. Therefore, Factor 1 was identified as a source indicative of fertilizer application, based on its characteristic element (Pb) and the supporting Pb isotope signature.

Ni (79.52%) and Cr (79.75%) were the main contributing elements of Factor 2. Cr was the most stable PTE in the weathering process of phosphatic rock (Fig. 2). Generally, the mobility of Cr in the environment was low, and the rock weathering was considered to be a significant source of Cr in soil (Duan et al., 2020; Sakizadeh & Zhang, 2021). Zhao et al. (2022a) and Zhao et al. (2022b) also stated that Cr and Ni in farmland soil were mainly derived from parent materials. Therefore, the most likely source of Factor 2 was phosphatic bedrock.

The main constituent PTE of Factor 3 in farmland soil were Cd (80.68%), As (66.47%) and Hg (83.49%). Studies have shown that pesticides had the ability to adsorb Cd, and the use of pesticides aggravated the level of Cd in farmland systems (Chiroma, Abdulkarim & Kefas, 2007; Undabeytia, Morillo & Maqueda, 1996). Meanwhile, the application of arsenic-containing pesticides was the main culprit of arsenic contamination in agricultural soils (Higgins, Metcalf & Robbins, 2022). Therefore, Factor 3 is interpreted as representing inputs from the use of agricultural pesticides and/or fungicides. This interpretation is strongly supported by the co-occurrence of Cd, As, and Hg—elements historically common in various pesticide formulations—and is consistent with the known ability of these chemicals to adsorb to soil particles and persist in the environment.

Factor 4 was classified as other sources, and the load of Cu and Zn on the factor was prominent, being 51.43% and 66.11%, respectively. Cu and Zn were relatively stable during weathering process (Fig. 2), and their content in farmland soil was similar to the background value (Table 1), indicating that Cu and Zn were less affected by human activities and were more likely to come from other natural sources. Therefore, the source class of factor 4 might be formed by the river and wind transport of weathering soils from other bedrock.

This study found that the use of fertilizers and pesticides in agricultural activities was the dominant factor causing severe contamination of Pb and Cd of surface soil in phosphatic zone. Integrating insights from weathering profiles and source apportionment clarifies the dynamics of cadmium (Cd) in the phosphatic zone. The weathering profiles demonstrate that phosphate bedrock weathering can lead to significant secondary enrichment of Cd in surface soils (‘PTE concentrations in the profiles’, Fig. 2), establishing a naturally elevated geogenic background. However, the PMF model indicates that the dominant source of Cd in the contemporary farmland topsoil is anthropogenic, linked to pesticide use (Fig. 6B, Factor 3). This suggests a paradigm where the high natural background predisposes the soil system to Cd accumulation, but recent and severe surface contamination is primarily driven by agricultural activities. The elevated bioavailability of Cd in farmland soil (‘Bioavailability of PTE in farmland soil’) further supports the significant overprint of an active, anthropogenic Cd pool on top of the pre-existing geogenic enrichment. Distinguishing between geogenic and pesticide-derived Cd is critical. Our multi-method approach provides several lines of evidence supporting the dominance of the latter in topsoil: (1) Source Apportionment (PMF): The PMF model explicitly isolated a factor (Factor 3) characterized by Cd, As, and Hg, a combination strongly indicative of historical pesticide use rather than bedrock geochemistry. (2) Isotopic and Spatial Decoupling: The Pb isotopic signature in topsoil was distinct from local bedrock and aligned with agricultural inputs (‘Pb isotopes’), demonstrating a clear anthropogenic overprint on surface geochemistry. While this is specific to Pb, it establishes the significant magnitude of agricultural disturbance, within which Cd inputs would also occur. (3) Contrasting Bioavailability/Mobility: The highly mobile nature of Cd in farmland soil (‘Bioavailability of PTE in farmland soil’) contrasts with the more residual character of purely geogenic elements (*e.g.*, Cr, Ni from Factor 2). Pesticide-derived Cd is often in more soluble and bioavailable forms compared to Cd incorporated within mineral lattices from weathering. (4) Mass Balance Consideration: The severe pollution level (87% of samples seriously polluted) and spatial heterogeneity of Cd in topsoil far exceed what would be expected from uniform, natural weathering enrichment alone, which typically creates an elevated but more spatially consistent background. The strong association with agricultural land use points to a recent, active input source.

### Consideration of contextual and potential confounding factors

The interpretation of PTE dynamics in our study area is set within a context of natural and anthropogenic variability. We recognize that factors such as local bedrock heterogeneity, karst topography, and differences in agricultural management practices could act as potential confounders. While our sampling design did not explicitly stratify for topography or collect detailed farm management records, several aspects of our study provide relevant context: (1) The characterization of multiple bedrock samples and the use of Al-normalized mass transfer coefficients (*τ*) help to distinguish geogenic weathering trends from simple inherited bedrock variability. (2) Although not stratified, our broad spatial distribution of farmland sampling sites across the region likely captures a range of topographic positions integrated within the “farmland” land-use type. (3) Variations in agricultural intensity are partially reflected in the measured soil properties (*e.g.*, pH, SOM, P_2_O_5_), which were analyzed for their correlation with PTE bioavailability. However, unmeasured fine-scale variations in these factors may contribute to residual variability in our dataset and represent a source of uncertainty, particularly in generalizing the exact magnitude of source contributions to other settings with different topographic or management contexts.

### Global context and implications

Placing our findings within a global context underscore both commonalities and unique aspects of PTE dynamics in phosphate-rich regions. Similar to the well-documented Gafsa-Metlaoui basin in North Africa (Khelifi et al., 2021), our study confirms that phosphate geology inherently leads to elevated environmental background levels of Cd and other PTEs, posing a universal baseline challenge. However, a key distinction emerges in the dominant anthropogenic pressure. In the North African context, intensive mining and ore processing are often the primary drivers of severe contamination and health risk *via* airborne particulate matter. In contrast, our results highlight that in the agriculturally dominated phosphate zone of southwestern China, the synergistic interplay between high geogenic background and intensive fertilizer/pesticide use becomes the critical risk multiplier, with the soil-crop pathway being paramount. This comparison suggests that management strategies must be region-specific: in mining districts, emission and dust control are priorities, whereas in agricultural phosphate zones, optimizing fertilizer/pesticide management and implementing *in-situ* soil immobilization (*e.g.*, using iron-based amendments as indicated by our bioavailability controls) are likely more effective. Future integrative studies that combine geogenic background assessment with source apportionment, as performed here, will be crucial for developing tailored mitigation policies in different types of phosphate provinces worldwide.

### Strengths and limitations

This study provides a comprehensive analysis of PTE dynamics in the phosphatic zone by integrating the investigation of both natural weathering processes and anthropogenic influences. A key strength lies in the combined application of Pb isotope tracing and the PMF model, which successfully differentiated between geogenic and agricultural sources. Furthermore, the examination of PTE geochemical fractions in weathered profiles offered crucial insights into their mobility and potential ecological risks, highlighting Cd’s significant bioavailability. The study has limitations. The source apportionment relies on receptor modeling and correlation analyses, which indicate but do not conclusively prove specific agricultural sources. First, the absence of direct chemical composition data for locally used fertilizers and pesticides means that our source apportionment for agricultural inputs relies on inference rather than direct analytical matching. Second, the findings on PTE enrichment and mobility are specific to the karst setting studied; their generalizability to other heterogeneous karst regions with differing geomorphology or land-use intensity may be limited. Future research incorporating direct analysis of these agricultural amendments and conducted across diverse karst terrains would strengthen the source identification and enhance the broader applicability of the findings. Additionally, the focus on total content and M3-extractable bioavailability could be expanded to include *in-situ* plant uptake studies for a more direct assessment of ecological and health risks.

## Conclusions

This study was guided by the need to quantify the interactions between geogenic and anthropogenic sources of PTEs in a phosphate-rich agricultural zone. By integrating analyses of weathering profiles and farmland soils, it is provided the following answers to the research questions.

(1) Regarding PTE behavior during weathering: The weathering of phosphate bedrock acts as a significant geogenic source, leading to substantial surface enrichment of Cd and Pb. This enrichment is mechanistically explained by the loss of calcium (providing exchange sites) coupled with secondary adsorption onto Fe/Al (hydr)oxides during pedogenesis, rather than simple inheritance.

(2) Regarding sources in topsoil: Quantitative source apportionment (PMF) combined with Pb isotope tracing demonstrates that agricultural activities, specifically phosphate fertilizer and pesticide application, are the dominant contemporary sources of Pb and Cd, respectively, in farmland topsoil. This anthropogenic input is superimposed upon and exacerbates the pre-existing high geogenic background.

(3) Regarding bioavailability control: Iron oxide content emerged as the primary soil property controlling PTE bioavailability, indicating that *in-situ* immobilization strategies using iron-based amendments could be effective for mitigating risks in this region.

Therefore, it is concluded that the severe PTE pollution in the phosphatic zone results from a synergistic coupling: a high natural background from active bedrock weathering primes the environment, while intensive agricultural inputs provide the immediate and dominant contaminant flux. This study underscores that effective management in such settings requires strategies that address both the legacy of geology and the pressures of modern agriculture.

## Supplemental Information

10.7717/peerj.21110/supp-1 Supplemental Information 1 Raw data

10.7717/peerj.21110/supp-2 Supplemental Information 2 Additional data, methodological details, and extended analyses

## Additional Information and Declarations

### Data Availability

The following information was supplied regarding data availability: The raw data is available in the Supplemental Files.
