# Supplementary material for: Phosphatic rock weathering and agricultural activities driving the dynamics of potentially toxic elements in surface soil of weathered zone
Source: PeerJ. 2026 Apr 20;14:e21110. doi: 10.7717/peerj.21110 (PMC13105190; doi:10.7717/peerj.21110)
Supplement: Supplemental Information 2 [file peerj-14-21110-s002.docx]

<Supplementary Materials>

**Phosphatic rock weathering and agricultural activities driving the dynamics of potentially toxic elements in surface soil of weathered zone**

Haiyan Zhang ^1, 2, a, *^, Changguang Liao ^1, 3, a^, Chao Wang ^1^, Xinggang Ye ^1^, Bo Du ^4^, Ying Yu ^1^, Lei Zhang ^5,^ ^[[1]](#footnote-1)^*

^1^ College of Ecology and Environment, Hubei Industrial Polytechnic, Shiyan, Hubei, China

^2^ College of Environment, Hohai University, Nanjing, Jiangsu, China

^3^ Bioengineering College, Chongqing University, Chongqing Municipality, China

^4^ China Resources Double-crane Pharmaceutical Co.,Ltd., Beijing Municipality, China

^5^ Nanjing Institute of Environmental Science, Ministry of Ecology and Environment Peoples Republic of China, Nanjing, Jiangsu, China

**Text S1 Sodium acetate-flame photometric method**

5.0 g of air-dried soil was weighed into a 50 mL centrifuge tube, shaken with 33 mL of 1 mol/L NaOAc solution, centrifuged, and then washed with 95% ethanol to remove the excess Na^+^, exchange of Na^+^ with 1 mol/L NH_4_OAc, and then the Na^+^ content was measured by flame photometer.

**Text S2 Speciation analysis of heavy metals in soil samples**

The geochemical fraction determination of heavy metals in soil was implemented by the three-step sequential extraction method proposed by the European Standard Reference Bureau (BCR method), which was divided into four fractions: weak acid soluble fraction, reducible fraction, oxidizable fraction and residual fraction (Memoli et al. 2018).

(1) Weak acid soluble fraction (F1): 1.00 g soil sample was weighed into a 100 ml centrifuge tube. The centrifuge tube was shook with 40 ml 0.11mol/L HAc solution at (22 ± 5)℃ for 16 h, then centrifuged at 3000 r/min for 20 min. After centrifugation, the supernatant was detected.

(2) Reducible fraction (F2): 40 mL of 0.5mol/L NH_2_·HCl solution was added to the residue after the first step treatment and shook at (22±5)℃ for 16 h, then centrifuged at 3000 r/min for 20 min. The supernatant was detected.

(3) Oxidizable fraction (F3): 10 ml 8.8mol/L H_2_O_2_ was added to the residue after the second step treatment and shook for 1 h at room temperature, then digested at (85 ± 2)℃ until the solution volume was reduced to 3 ml. A second 10 ml of H_2_O_2_ was added, the mixture was digested for 1 h at 85 ± 2 °C. When 1 ml of the solution was left, 50 ml 1.0mol/L NH_4_Ac solution was added to the wet-cooled residue, and shook at (22 ± 5)℃ for 16 h, then centrifuged at 3000 r/min for 20 min. The supernatant was detected.

(4) Residue fraction (F4): 0.10 g of the residue was weighed into a crucible after the third step and soaked with a little water, then digested with a mixture of 3.0 mL HCl, 2.0 mL HNO_3_, 1.0 mL HClO_4_ and 5.0 mL HF on an electric heat plate until without white smoke. 1 mL 6 mol/L HCl was added to the crucible, and heated to dissolve the solids. Finally, the solution was cooled and diluted to 10 mL with deionized water for detection.

The accuracy of the sequential extraction procedure was evaluated by comparing the sum of the four extracted fractions (F1+F2+F3+F4) with the independently measured total concentration of each element. The average recoveries for all analyzed PTEs ranged from 85% to 110%, which is within the acceptable range for this method. Certified reference materials for soils (e.g., BCR-701) were processed in parallel with each batch of samples to monitor procedural accuracy.

**Text S3: Detailed procedure for Mehlich-3 extraction**

The bioavailable fraction of PTEs was extracted using the Mehlich-3 (M3) solution. Briefly, 2.5 g of air-dried soil (<2 mm) was weighed into a 50 mL centrifuge tube. 25 mL of the M3 extractant (composition as stated in the main text) was added, resulting in a soil: extractant ratio of 1:10 (w/v). The mixture was shaken on an end-over-end shaker at 180 rpm for 5 minutes at room temperature (25 ± 2°C). Immediately after shaking, the suspension was filtered through a 0.45-μm membrane filter. The filtrate was acidified with HNO₃ and stored at 4°C prior to analysis.

**Text S4: Instrumental analysis and quality assurance/quality control (QA/QC)**

All sample analyses followed strict quality assurance and quality control (QA/QC) protocols. The total concentrations of potentially toxic elements (PTEs) were determined using inductively coupled plasma mass spectrometry (ICP-MS, IRIS Advantage, Thermo Jarrell Ash, USA) for Cd, Pb, Cu, Zn, Ni, and Cr, and atomic fluorescence spectrometry (AFS, AFS-2100, China) for Hg and As. Calibration was performed using multi-element standard solutions from the National Center for Reference Materials (China), with verification at the start of each analytical batch and after every 20 samples. Method detection limits (MDLs), determined as three times the standard deviation of seven replicate analyses of a procedural blank, are provided in Table S5. Accuracy was monitored by processing certified reference materials (GSS-5 and GSS-8 soil CRMs) in parallel with each batch of 20 samples, yielding average recoveries of 88–112% for all elements. For the sequential extraction procedure, accuracy was further assessed by comparing the sum of the four extracted fractions to the total concentration, with recoveries ranging from 85% to 110%. Precision was controlled by analyzing duplicate samples (one duplicate per 10 samples), with relative standard deviations typically below 10%. Procedural blanks, included in each digestion batch (one blank per 10 samples), consistently showed values below the respective MDLs. To manage potential batch effects, samples from the same profile or geographic cluster were analyzed within the same batch, and calibration was verified using a mid-range standard within each batch.

For Pb isotope analysis, measurements were conducted on a Nu Plasma II multi-collector ICP-MS (MC-ICP-MS) equipped with an Aridus II desolvating nebulizer. Instrumental mass bias was corrected using standard-sample bracketing with the NIST SRM 981 standard and external normalization with a Tl spike (NIST SRM 997, assuming ${}^{205}\text{Tl}/^{203}\text{Tl}=2.3885$). Analytical accuracy and long-term reproducibility were verified through the concurrent analysis of USGS rock reference materials (BCR-2, BHVO-2, AGV-2, RGM-2). The measured isotopic ratios for these reference materials agreed well with certified values. The long-term external reproducibility (2SD) for ${}^{206}\text{Pb}/^{207}\text{Pb}$ and ${}^{208}\text{Pb}/^{206}\text{Pb}$ ratios, based on repeated measurements of NIST SRM 981, was better than 0.01.

**Table captions**

Table S1. Pollution classification of soil-bound heavy metals based on pollution index

Table S2. The mean concentrations of PTE in phosphatic bedrock and topsoil of weathered profiles

Table S3. Correlation matrix between soil properties and ratio of extractable-PTE using Mehlich3 extractant.

Table S4. Pb isotope ratios of different sample types

Table S5. Method detection limits (MDLs), quality control parameters, and uncertainty estimates for the analysis of potentially toxic elements (PTEs)

Table S6. Bootstrap runs results of PMF

Table S1. Pollution classification of soil-bound heavy metals based on pollution index

| Classification | single pollution index (*P_i_*) | pollution classification |
| --- | --- | --- |
| Ⅰ | *P_i_*＜1 | clean |
| Ⅱ | 1 ≤ *P_i_*＜ 2 | Slightly polluted |
| Ⅲ | 2 ≤ *P_i_*＜ 3 | moderately polluted |
| Ⅳ | *P_i_*≥ 3 | Seriously polluted |

Table S2. The mean concentrations of PTE in phosphatic bedrock and topsoil of weathered profiles

| PTE (mg/kg) | Cd | Pb | Cu | Zn | Ni | Cr | As | Hg |
| --- | --- | --- | --- | --- | --- | --- | --- | --- |
| Phosphatic bedrock | 0.07 | **78** | 22.9 | 71 | 28.1 | 61 | **21** | **0.47** |
| UCC ^a^ | 0.09 | 17 | 28 | 67 | 47 | 92 | 4.8 | 0.05 |
| Topsoil of weathered profiles | **0.23** | **123** | **34.7** | **98** | **39.8** | **75** | **28** | **0.5** |
| Background of China ^a^ | 0.097 | 26 | 22.6 | 74.2 | 26.9 | 61 | 11.2 | 0.065 |

^a^ (Zhao et al. 2018)

Table S3. Correlation matrix between soil properties and Bio-PTE, showing Pearson correlation coefficients (r) with their 95% confidence intervals (95% CI) and significance levels.

|  | Soil properties | | | | | | |
| --- | --- | --- | --- | --- | --- | --- | --- |
|  | pH (r, 95%CI) | SOM (r, 95%CI) | CEC (r, 95%CI) | P_2_O_5_ (r, 95%CI) | Al_2_O_3_ (r, 95%CI) | TFe_2_O_3_ (r, 95%CI) | SiO_2_ (r, 95%CI) |
| Bio-Cd | -0.480**  ( -0.589, -0.354 ) | 0.004  ( -0.138, 0.146 ) | 0.033  ( -0.109, 0.174 ) | -0.307**  ( -0.432, -0.172 ) | -0.174*  ( -0.305, -0.037 ) | -0.131  ( -0.264, 0.006 ) | -0.271**  ( -0.399, -0.134 ) |
| Bio-Pb | -0.504**  ( -0.608, -0.383 ) | -0.117  ( -0.257, 0.027 ) | -0.083  ( -0.224, 0.061 ) | -0.259**  ( -0.387, -0.121 ) | -0.264**  ( -0.392, -0.127 ) | -0.346**  ( -0.466, -0.214 ) | -0.496**  ( -0.601, -0.374 ) |
| Bio-Cu | 0.094  (-0.048, 0.233) | 0.217**  (0.078, 0.349) | 0.244**  (0.106, 0.374) | -0.113  (-0.253, 0.031) | -0.236**  (-0.367, -0.098) | -0.286**  (-0.415, -0.150) | -0.177*  (-0.308, -0.040) |
| Bio-Zn | -0.354**  (-0.475, -0.222) | -0.016  (-0.158, 0.126) | -0.078  (-0.219, 0.066) | -0.193*  (-0.323, -0.056) | -0.038  (-0.179, 0.104) | -0.047  (-0.187, 0.095) | 0.035  (-0.107, 0.176) |
| Bio-Ni | -0.137  (-0.270, 0.000) | 0.141  (0.000, 0.277) | 0.203*  (0.064, 0.335) | -0.096  (-0.235, 0.046) | -0.200*  (-0.332, -0.061) | -0.063  (-0.203, 0.079) | 0.068  (-0.074, 0.208) |
| Bio-Cr | -0.056  (-0.197, 0.087) | 0.002  (-0.140, 0.144) | 0.082  (-0.059, 0.221) | -0.067  (-0.207, 0.075) | -0.109  (-0.249, 0.034) | -0.280**  (-0.410, -0.143) | -0.059  (-0.200, 0.084) |
| Bio-As | -0.075  (-0.215, 0.068) | 0.003  (-0.139, 0.145) | -0.006  (-0.148, 0.136) | 0.009  (-0.133, 0.151) | -0.115  (-0.255, 0.028) | -0.569**  (-0.665, -0.458) | 0.125  (-0.018, 0.264) |
| Bio-Hg | -0.341**  (-0.463, -0.208) | -0.093  (-0.232, 0.049) | -0.124  (-0.263, 0.019) | 0.038  (-0.104, 0.179) | -0.137  (-0.270, 0.000) | -0.269**  (-0.397, -0.132) | -0.191*  (-0.321, -0.054) |

A single asterisk indicates a significant correlation (*P*＜0.05), and a double asterisk indicates a extremely significant correlation (*P*＜0.01).

Table S4. Pb isotope ratios of different sample types

| Sample types | ^206^Pb/^207^Pb | | | | ^208^Pb/^206^Pb | | | |
| --- | --- | --- | --- | --- | --- | --- | --- | --- |
|  | Min | Max | Mean | SD | Min | Max | Mean | SD |
| Farmland soils in PMA | **1.172** | **1.213** | **1.187** | **0.022** | **2.002** | **2.109** | **2.061** | **0.044** |
| Phosphatic bedrock | **1.253** | **1.716** | **1.421** | **0.21** | **1.362** | **1.928** | **1.721** | **0.25** |
| Natural sources in China ^a^ | 1.190 | 1.373 | 1.224 | 0.057 | 1.802 | 2.085 | 2.031 | 0.089 |
| Coal in SW ^a^ | 1.183 | 1.383 | 1.218 | 0.037 | 1.791 | 2.117 | 2.057 | 0.058 |
| Vehicle exhaust ^b^ | 1.150 | 1.162 | 1.156 | 0.005 | 2.106 | 2.115 | 2.111 | 0.004 |
| Fertilizer ^c^ | 1.199 | 1.222 | 1.211 | 0.009 | 2.011 | 2.024 | 2.016 | 0.006 |
| Rainwater ^b^ | 1.165 | 1.173 | 1.170 | 0.002 | 2.100 | 2.106 | 2.103 | 0.002 |
| Atmospheric deposition ^c^ | 1.112 | 1.140 | 1.126 | 0.011 | 2.126 | 2.139 | 2.133 | 0.005 |

^a^ (Bi et al. 2017); ^b^ (Zhao et al. 2015); ^c^ (Liu et al. 2019)

Table S5. Method detection limits (MDLs), quality control parameters, and uncertainty estimate for the analysis of potentially toxic elements (PTEs)

| Element | Method Detection Limit (MDL, mg/kg) | Certified Reference Material (CRM) | Average Recovery ± Standard Deviation (%) | Number of Replicates (Duplicates per batch) | Relative Standard Deviation (RSD, %) of Duplicates | Primary Analytical Technique |
| --- | --- | --- | --- | --- | --- | --- |
| Cd | 0.01 | GSS-5 | 95 ± 5 | 1 per 10 samples | < 8 | ICP-MS |
| Pb | 0.1 | GSS-5 | 102 ± 4 | 1 per 10 samples | < 5 | ICP-MS |
| Cu | 0.2 | GSS-5 | 98 ± 6 | 1 per 10 samples | < 6 | ICP-MS |
| Zn | 0.5 | GSS-8 | 105 ± 7 | 1 per 10 samples | < 7 | ICP-MS |
| Ni | 0.5 | GSS-8 | 92 ± 8 | 1 per 10 samples | < 10 | ICP-MS |
| Cr | 1 | GSS-8 | 96 ± 9 | 1 per 10 samples | < 9 | ICP-MS |
| As | 0.05 | GSS-5 | 101 ± 6 | 1 per 10 samples | < 8 | AFS |
| Hg | 0.002 | GSS-5 | 88 ± 10 | 1 per 10 samples | < 12 | AFS |

Notes: MDL: Determined as three times the standard deviation of seven procedural blank analyses; CRM: Soil certified reference materials GSS-5 and GSS-8 (China National Standard Samples) were used for accuracy control; Recovery: Mean recovery and its standard deviation from CRM analyses across all analytical batches; Replicates: One duplicate sample was analyzed for every ten field samples to monitor precision; RSD: The typical range of Relative Standard Deviation observed for duplicate analyses; Technique: ICP-MS (Inductively Coupled Plasma Mass Spectrometry); AFS (Atomic Fluorescence Spectrometry).

Table S6. Bootstrap runs results of PMF

| Factor 1 | Base Run | Bootstrap Runs | | | | | | |
| --- | --- | --- | --- | --- | --- | --- | --- | --- |
| Species | Profile | Mean | Std. Dev. | 5th | 25th | Median | 75th | 95th |
| Cd | 0.11593 | 0.117615 | 0.09696 | 0 | 0.048454 | 0.10181 | 0.175398 | 0.280994 |
| Pb | 109.1 | 110.8905 | 12.67716 | 93.6195 | 103.1325 | 111.295 | 121.1825 | 128.763 |
| Cu | 5.4539 | 9.174193 | 2.856075 | 3.25458 | 7.487375 | 9.6587 | 11.2195 | 12.7459 |
| Zn | 0.14806 | 20.99985 | 13.54527 | 0 | 10.89775 | 22.4175 | 31.5145 | 42.41215 |
| Ni | 2.2139 | 2.311818 | 2.772822 | 0 | 0 | 0.00021 | 2.902125 | 10.04191 |
| Cr | 19.799 | 10.65434 | 9.944998 | 0 | 2.44545 | 9.95895 | 15.383 | 29.57725 |
| As | 2.92E-16 | 0.659482 | 0.637956 | 0 | 0 | 0.192155 | 0.964653 | 2.351185 |
| Hg | 0 | 0.002108 | 0.002671 | 0 | 0 | 0 | 0.001545 | 0.015589 |
| Factor 2 | Base Run | Bootstrap Runs | | | | | | |
| Species | Profile | Mean | Std. Dev. | 5th | 25th | Median | 75th | 95th |
| Cd | 0.033729 | 0.024391 | 0.025793 | 0 | 0 | 0 | 0.032293 | 0.133356 |
| Pb | 1.9214 | 5.865965 | 5.719468 | 0 | 0 | 0.16638 | 10.6255 | 22.9746 |
| Cu | 0 | 3.02444 | 2.657294 | 0 | 0 | 1.7492 | 4.79515 | 9.8309 |
| Zn | 15.075 | 21.93853 | 20.87502 | 0 | 0.07273 | 18.869 | 33.1585 | 62.9413 |
| Ni | 40.819 | 32.04446 | 12.2909 | 5.9279 | 27.1885 | 35.799 | 40.6145 | 46.0908 |
| Cr | 101.91 | 80.49513 | 26.16877 | 19.5291 | 70.3665 | 88.273 | 99.8005 | 107.207 |
| As | 3.4393 | 3.218078 | 2.041477 | 0 | 1.8082 | 3.2128 | 4.43835 | 6.76453 |
| Hg | 0.005305 | 0.005747 | 0.005263 | 0 | 4.11E-05 | 0.004846 | 0.007396 | 0.019495 |
| Factor 3 | Base Run | Bootstrap Runs | | | | | | |
| Species | Profile | Mean | Std. Dev. | 5th | 25th | Median | 75th | 95th |
| Cd | 0.62488 | 0.528061 | 0.18181 | 0.084416 | 0.517215 | 0.577825 | 0.646128 | 0.694413 |
| Pb | 25.859 | 15.55221 | 14.07814 | 0 | 0 | 13.1295 | 26.98675 | 39.33165 |
| Cu | 3.4149 | 3.358366 | 2.433258 | 0 | 0.741728 | 2.27 | 4.7214 | 11.5879 |
| Zn | 15.236 | 10.31487 | 9.40704 | 0 | 0 | 6.1172 | 15.16775 | 36.95725 |
| Ni | 1.69E-10 | 3.185796 | 2.965384 | 0 | 0 | 0.339805 | 4.251 | 14.9672 |
| Cr | 2.1265 | 8.720227 | 8.33294 | 0 | 0 | 4.31435 | 13.3635 | 32.32955 |
| As | 9.9908 | 8.130368 | 3.551698 | 0.978888 | 6.0642 | 8.9188 | 10.5605 | 12.84815 |
| Hg | 0.026819 | 0.021405 | 0.011133 | 0.001722 | 0.012649 | 0.022925 | 0.028272 | 0.038556 |
| Factor 4 | Base Run | Bootstrap Runs | | | | | | |
| Species | Profile | Mean | Std. Dev. | 5th | 25th | Median | 75th | 95th |
| Cd | 0 | 0.071692 | 0.067546 | 0 | 0.001258 | 0.057287 | 0.10529 | 0.239768 |
| Pb | 11.311 | 15.44997 | 12.99231 | 0 | 5.6682 | 12.616 | 24.099 | 39.6982 |
| Cu | 9.3919 | 4.271425 | 3.982653 | 0 | 1.62E-14 | 1.1897 | 8.2061 | 13.2166 |
| Zn | 59.404 | 56.65735 | 19.93876 | 30.2376 | 44.343 | 54.451 | 70.788 | 93.8204 |
| Ni | 8.2984 | 9.544001 | 6.84106 | 0.047396 | 4.0554 | 9.0192 | 12.947 | 22.5114 |
| Cr | 3.9457 | 12.36057 | 11.51409 | 0 | 0.6432 | 9.6251 | 18.16 | 40.9898 |
| As | 1.5995 | 2.141009 | 1.95167 | 0 | 0.36834 | 1.677 | 3.1215 | 6.54552 |
| Hg | 0 | 0.002696 | 0.001932 | 0 | 0 | 0 | 0.000737 | 0.025909 |

**Figure captains**

Fig. S1. Pearson correlation coefficient among the “τ” values of PTE, calcium oxide and Fe-Al oxides in weathered profiles

Fig. S2. A schematic linking isotope fields to putative PMF factors


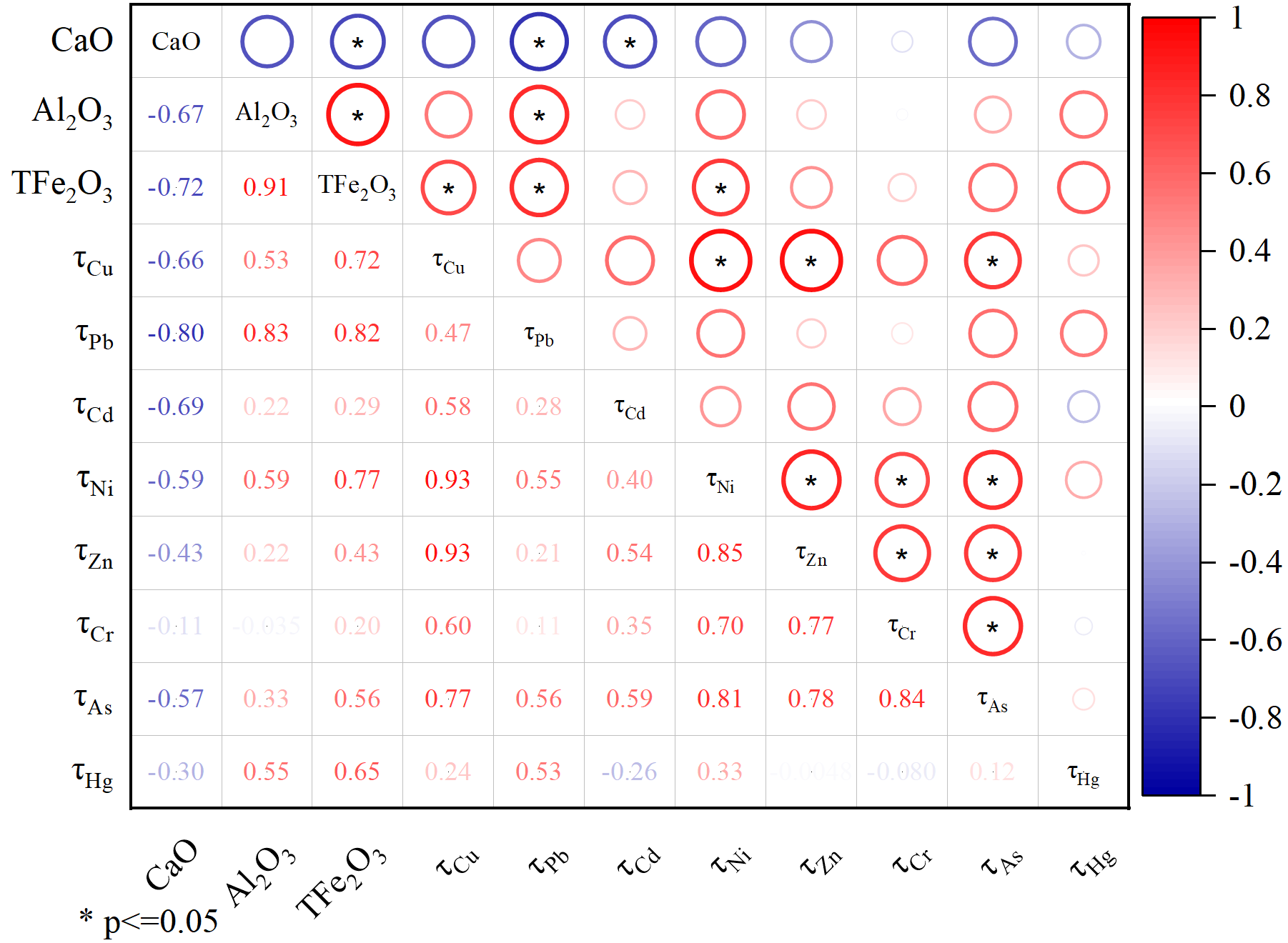


Fig. S1. Pearson correlation coefficient among the “τ” values of PTE, calcium oxide and Fe-Al oxides in weathered profiles

**
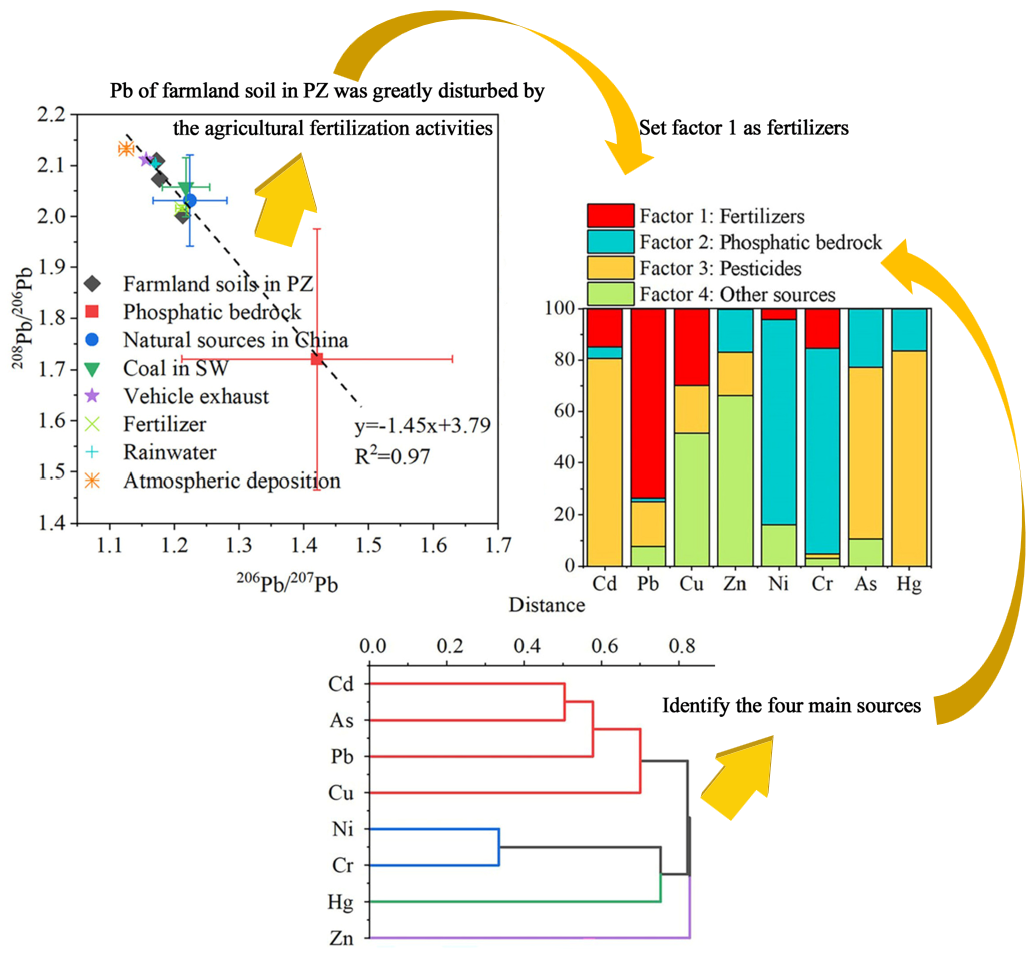
**

Fig. S2. A schematic linking isotope fields to putative PMF factors

**References**

Bi, X., Z. Li, S. Wang, L. Zhang, R. Xu, J. Liu, H. Yang & M. Guo (2017) Lead Isotopic Compositions of Selected Coals, Pb/Zn Ores and Fuels in China and the Application for Source Tracing. *Environmental Science & Technology,* Vol.51**,** 13502-13508.

Liu, J., D. Wang, B. Song, Z. Chen, X. Zhang & Y. Tang (2019) Source apportionment of Pb in a rice-soil system using field monitoring and isotope composition analysis. *Journal of Geochemical Exploration,* 204**,** 83-89.

Memoli, V., E. Eymar, C. García-Delgado, F. Esposito, S. C. Panico, A. De Marco, R. Barile & G. Maisto (2018) Soil element fractions affect phytotoxicity, microbial biomass and activity in volcanic areas. *Science of the Total Environment,* 636**,** 1099-1108.

Zhao, W. F., Y. X. Song, D. X. Guan, Q. Ma, C. Guo, Y. B. Wen & J. F. Ji (2018) Pollution status and bioavailability of heavy metals in soils of a typical black shale area. *Journal of Agro-Environment Science,* 37**,** 1332-1341.

Zhao, Z., W. Zhang, X. Li, Z. Yang, H. Zheng, H. Ding, Q. Wang, J. Xiao & P. Fu (2015) Atmospheric lead in urban Guiyang, Southwest China: Isotopic source signatures. *Atmospheric Environment,* 115**,** 163-169.

1. *** Corresponding author:**

   Lei Zhang ^5^

   No.8, Jiangwang Miao Street, Nanjing City, Jiangsu Province, 210042, PR China

   Email address: [lizaoyutian@126.com](mailto:lizaoyutian@126.com)

   Haiyan Zhang ^1, 2^

   No.38, Beijing Middle Road, Shiyan City, Hubei Province, 442000, PR China

   Email address: [hhu_zhy@163.com](mailto:hhu_zhy@163.com)

   ^a^ These authors contributed equally to this work. [↑](#footnote-ref-1)
